# Effect of Transcranial Direct Current Stimulation on the Mismatch Negativity Features of Deviated Stimuli in Children With Autism Spectrum Disorder

**DOI:** 10.3389/fnins.2022.721987

**Published:** 2022-02-09

**Authors:** Changcheng Sun, Zhuoyue Zhao, Longlong Cheng, Rong Tian, Wenchang Zhao, Jingang Du, Ying Zhang, Chunfang Wang

**Affiliations:** ^1^Academy of Medical Engineering and Translational Medicine, Tianjin University, Tianjin, China; ^2^Department of Rehabilitation Medical, Tianjin Union Medical Centre, Tianjin, China; ^3^China Electronics Cloud Brain (Tianjin) Technology Co., Ltd., Tianjin, China

**Keywords:** autism spectrum disorder (ASD), transcranial direct current stimulation (tDCS), EEG, mismatch negativity (MMN), dorsolateral prefrontal cortex (DLPFC)

## Abstract

Autism spectrum disorder (ASD) is a devastating mental disorder in children. Currently, there is no effective treatment for ASD. Transcranial direct current stimulation (tDCS), which is a non-invasive brain stimulation neuromodulation technology, is a promising method for the treatment of ASD. However, the manner in which tDCS changes the electrophysiological process in the brain is still unclear. In this study, we used tDCS to stimulate the dorsolateral prefrontal cortex area of children with ASD (one group received anode tDCS, and the other received sham tDCS) and investigated the changes in evoked EEG signals and behavioral abilities before and after anode and sham stimulations. In addition to tDCS, all patients received conventional rehabilitation treatment. Results show that although conventional treatment can effectively improve the behavioral ability of children with ASD, the use of anode tDCS with conventional rehabilitation can boost this improvement, thus leading to increased treatment efficacy. By analyzing the electroencephalography pre- and post-treatment, we noticed a decrease in the mismatch negativity (MMN) latency and an increase in the MMN amplitude in both groups, these features are considered similar to MMN features from healthy children. However, no statistical difference between the two groups was observed after 4 weeks of treatment. In addition, the MMN features correlate well with the aberrant behavior checklist (ABC) scale, particularly the amplitude of MMN, thus suggesting the feasibility of using MMN features to assess the behavioral ability of children with ASD.

## Introduction

Autism spectrum disorder (ASD) is a biologically based neurodevelopmental disorder (developmental disability) that is defined by the following diagnostic criteria: deficits in social communication and interaction and presence of restricted, repetitive patterns of behavior, interests, or activities that can persist throughout life (Battle, 2013; Principi and Esposito, 2020). The population of children with ASD is generally large; approximately 1 in 54 children has been identified with ASD in the United States in 2016 (Maenner et al., 2020). There are no exact statistics on the number of patients with ASD in China. Some scholars believe that the actual number may reach 2.6–8 million. Worryingly, the prevalence of ASD is increasing year by year, with the United States experiencing an increase of 150% from 2000 to 2014. This situation is more severe in China, which has a rate of increase of 200,000 ASD cases per year. Therefore, the increasing incidence of ASD is a problem that needs the attention of the global community.

Different factors could increase the susceptibility of a child to develop ASD, including environmental, biological, genetic, pregnancy-related, and behavioral factors (Hallmayer et al., 2011; Anagnostou et al., 2014; Gesundheit and Rosenzweig, 2017). The factor with the highest probability is genetic deficits, but susceptibility to ASD is also correlated with the living environment. The exact cause of ASD remains unclear. Several interventions have been investigated and developed for ASD, including behavioral intervention therapy, drug therapy, psychological analysis, sensory integration therapy, and assistive technology. Most of these therapies originate from the perspectives of psychology and behavior and achieve treatment and relief by correcting the abnormal behavior of children with ASD or by changing their psychological state. Currently, no treatment has been shown to cure ASD or eliminate its core symptoms.

Most researchers agree that ASD is a cognitive developmental disorder caused by impaired brain function (Miyahara, 2013; Jutla et al., 2021). Dickinson et al. (2016) and Lee et al. (2017) showed the imbalances between excitation and inhibition in synaptic transmission and neural circuits in patients with ASD Anatomically, patients with ASD have more cortical mini-columns and smaller volumes than healthy individuals, particularly in the dorsolateral prefrontal cortex (DLPFC) area (Casanova, 2006; Casanova et al., 2006). Physical stimulation regulates the balance between neuronal excitation and inhibition in the nerve conduction circuit and may play a positive role in the treatment of ASD. At present, researchers have investigated various detection and repair technologies for the impairment of cognitive function in the central nervous system. Transcranial direct current stimulation (tDCS) is a non-invasive brain stimulation technique that uses weak direct current to induce changes in intracortical excitability. The direction of these modulations depends on stimulation polarity: Anodal stimulation increases excitability, whereas cathodal stimulation diminishes excitability (Vaseghi et al., 2015). The main advantages of tDCS are safety, stability, non-invasiveness, and ease of clinical application. tDCS is widely used in neurological and psychiatric diseases, such as stroke, depression, and Parkinson’s disease. It has also been studied in attention-deficit hyperactivity disorder (ADHD), cerebral palsy, and language disorders, and no serious adverse reactions have been reported.

Electroencephalography (EEG) is a non-invasive method for recording the electrical activity of the brain along the scalp and is a powerful tool for studying complex neuropsychiatric disorders (Kang et al., 2018). In addition to EEG, functional magnetic resonance imaging (fMRI), which is an important neuroimaging detection method, has also been applied in many studies. However, considering that the subjects of the current study were children with ASD, it is difficult to complete long-term data collection in a small space with nuclear magnetic equipment. Therefore, the changes in evoked EEG signals and behavioral abilities of each child were investigated in the current study. Event-related potential (ERP) is an evoked potential with phase-locked time-specific events, which can reflect the neurophysiological changes of the brain during cognitive processes. When the nervous system receives a specific stimulus, relevant EEG waveforms can be detected in the corresponding regions of the brain, such as P1, N1, P2, P3, and mismatch negativity (MMN). The MMN is a change-specific component of auditory ERP, which is evoked by a discriminable change in any physical feature of a frequently presented stimulus (Čeponiene et al., 1998; Näätänen et al., 2004). MMN is generally considered an endogenous ERP component that represents an automatic neuronal change-detection process. In addition, MMN can be measured in an unconscious situation and does not require the patient to take the initiative to participate. It is more suitable for the evaluation of children with ASD who cannot concentrate. Therefore, it is now widely used in clinical applications for the diagnosis of certain neurological deficits. Relevant studies have shown that compared with healthy children, patients with ASD have robust MMN deficits, thus suggesting an altered central ability in auditory discrimination (Vlaskamp et al., 2017; Chen et al., 2020; Di Lorenzo et al., 2020). In the current study, a single-blind, sham-controlled experiment was designed for subjects with ASD. We placed the anode of tDCS over the left DLPFC and analyzed the changes in behavior and MMN features of children with ASD after 4 weeks of treatment to verify the clinical efficacy of tDCS.

## Materials and Methods

### Patient Demographics and Recruitment

Forty children with ASD (age range: 4–12 years; 33 males and 7 females) were recruited in this study from December 2018 to January 2020. The participants were all diagnosed with ASD by using the fifth edition of the *Diagnostic and Statistical Manual of Mental Disorders* (Battle, 2013), and the diagnoses were confirmed by clinical experts.

Participants were randomly assigned to the anodal tDCS stimulation group (atDCS group) or sham stimulation group (stDCS group), with 20 participants in each group. One participant dropped out of the study because of personal reasons, and two participants were excluded from the analysis because of excessive noise in the EEG recording. Therefore, we analyzed the data from 37 participants. At the end of this study, 19 participants (15 males and 4 females; mean ± SD age: 8.0 ± 1.9 years) received anodal tDCS stimulation, and 18 participants (15 males and 3 females; mean ± SD age: 7.6 ± 2.1 years) received sham stimulation. Most of the enrolled patients were accompanied by various degrees of comorbidities, including anxiety disorder, ADHD, attenuated psychosis syndrome (APS). As shown in Table 1, one child may have multiple comorbidities. All patients received conventional treatment methods (details shown in section “Conventional Rehabilitation Treatment”), in addition to tDCS intervention during the enrollment period, and were drug free for at least 2 weeks before the study began. There was no statistical difference between the two groups of participants in terms of gender, age, handiness, and comorbidities. This study was approved by the ethics committee of Tianjin Union Medical Center, Tianjin, China. All participants and their guardians provided written informed consent before participation.

**TABLE 1:** Number of comorbidities in the two groups.

Groups	Anxiety	ADHD	Learning disorder	Obsessive–compulsive	Oppositional defiant	Eating disorder	APS
atDCS	4	8	5	4	2	1	1
stDCS	3	9	5	4	2	0	1

### Transcranial Direct Current Stimulation Procedures

The tDCS device used in this study was an IS200 intelligent electrical stimulator produced by Sichuan Intelligent Electronic Industry Co., Ltd. The current was delivered through a pair of 35 cm^2^ sponge electrodes (i.e., the anode and cathode) that were soaked in sterile saline before the experiment to ensure good electrical conductivity. The anodal electrode was placed over the left DLPFC (F3 in the 10/20 system), and the cathode was placed over the right supraorbital area for all participants. For the anodal tDCS, stimulation current began with an 8 s ramp-up to 1.5 mA, lasted for 20 min at 1.5 mA, and ended with an 8 s ramp-down to 0 mA. For sham stimulation, the current increased at the same rate as the anodal stimulation at the beginning; the difference is that the stimulation current of 1.5 mA only lasted for 30 s, and no current was given after the first 30 s. Both the atDCS and stDCS groups had the same intervention duration (21 min). The 30 s fade-in and fade-out times were set because the procedure ensured that the participants felt a tingling sensation at the beginning of the stimulation. Transient EEG artifacts were observed only during the fade-in and fade-out phases of tDCS stimulation. A similar protocol was used in comparable studies (Kunze et al., 2016; Kang et al., 2018). The tDCS intervention was a single-blind experiment, and the patients did not know what kind of treatment they received. tDCS stimulation was performed for a total of 4 weeks for three times a week, and the two treatment sessions were separated by at least 1 day.

### Conventional Rehabilitation Treatment

There are many different manifestations in children with ASD, including social communication and behavioral and cognitive abilities. Therefore, treatment plans are usually multidisciplinary. We chose the rehabilitation treatment according to the child’s individual needs, including behavioral intervention strategies (Tiura et al., 2017), sensory integration therapy (Case-Smith et al., 2015), and speech therapy methods (Li et al., 2018). Table 2 shows the statistical results of the two groups of patients receiving different rehabilitation treatment items, and the combination of the types of treatment received by the patients was used as the evaluation index. The chi-square test was used for statistical analysis, and the results showed that there was no statistical difference between the two groups of patients. The treatment plan was the same as that for tDCS, which was also performed three times a week, and each treatment session lasted approximately 40 min.

**TABLE 2:** Statistical analysis of the treatment items received by the two groups.

Groups	b + s1	b + s2	b + s1 + s2
atDCS (*n* = 19)	11	2	6
stDCS (*n* = 18)	9	2	7
χ^2^	0.25		
*p*	0.882		

*b, behavioral intervention strategies; s1, sensory integration therapy; s2, speech therapy.*

### Behavioral Measures and Evaluation

In addition to EEG acquisition, we used the aberrant behavior checklist (ABC) to evaluate the behavior of children with ASD (Kat et al., 2020). The ABC scale covers almost all aspects of the symptoms of patients with ASD. It assesses five problem aspects, including communication, feeling, body movement, language, and self-care ability, and contains 57 sub-items. The assessment requires the cooperation of the participant’s guardians, who understand the child’s current situation. We mainly focused on children’s performance in the previous week.

To avoid bias caused by human subjective factors on the experiment results, the evaluation process of the scale adopted a double-blind method, i.e., participants and their guardians did not know whether they were in the atDCS stimulation group or the stDCS stimulation group. Furthermore, the therapist who evaluated the subject also did not know which group the participant was placed. At the same time, to ensure the accuracy of the assessment, all scale scores were completed under the guidance of an experienced therapist.

### Electroencephalography Recording and Processing

The EEG signals were recorded using an extended international 10/20 system with a Neuroscan 64-electrode EEG cap. Before starting the recording, the skin resistances of 64 channels were measured and maintained below 10 kΩ. All channels were measured against a common reference (Cz) with a sampling rate of 1,000 Hz. Eye movements were monitored with two pairs of electrodes: one pair above and below the left eye and another pair at the outer corner of the left and right eyes.

In this study, the MMN was induced using an auditory oddball task, and a series of standard auditory stimuli were interspersed with deviation tones. MMN was the negative EEG wave induced by the deviation stimulus minus the standard stimulus, which generally appears 100–250 ms after stimulus onset. The oddball task contained 4 types of auditory stimuli for a total of 1,000 times. The standard stimulus frequency was 1,000 Hz, the duration was 50 ms, the sound intensity was 60 db, and the probability of occurrence was 70%. There were three types of deviation stimuli: frequency deviation stimulation (FDS; 1,500 Hz, 50 ms, 60 dB, 10%), time deviation stimulation (TDS; 1,000 Hz, 100 ms, 60 dB, 10%), and time-frequency deviation stimulation (TFDS; 1,500 Hz, 100 ms, 60 dB, 10%). All auditory stimuli were standard sine waves with an intensity of 75 db. Stimuli were produced and delivered using the NeuroStim equipment. The auditory stimuli were presented through a loudspeaker, which was placed 1 m in front of the subjects. Each participant received 1,000 stimuli.

The EEG signals were re-referenced offline to bilateral mastoid electrodes (M1 and M2) at a 200 Hz sampling rate, after which the eye movement interference and baseline drift were removed. In all channels, except the four channels located on the eyes, if the standard deviation of EEG data in any 200 ms epoch was greater than 30 uV, it was considered that this segment of data might be affected by interference and caused excessive fluctuations. The epochs containing such segments were deleted in the following analysis. EEG epochs of −100 to 400 ms were extracted and averaged, and 0 ms denotes the start time when the stimulus is presented.

Before and after 4 weeks of treatment, behavioral ability assessment and EEG data collection were performed on all subjects.

### Statistical Analysis

Data are presented as the mean ± standard error of the mean ($\bar{\text{x}}$ ± s). The categorical variables (gender, handiness, and comorbidity) in the basic information of the two groups were compared using the chi-square test, and type I errors were corrected using the Bonferroni method. The discrete variables (age) were compared using one-way analysis of variance. To quantitatively evaluate changes in metrics across the pre- and post-tDCS intervals, the normality of data was first investigated using the Shapiro–Wilk test (*p* > 0.05). An independent sample *t*-test was used to compare the data changes before and after tDCS between the sham and anode groups, and a paired sample *t*-test was used to analyze the changes in the same group of subjects. Pearson’s correlation coefficients were used to examine the relationships between the changes in the scale evaluation results and the MMN component features.

## Results

### Behavioral Evaluations

The ABC scores post-tDCS to pre-tDCS were compared for the two groups. The *t*-test returned normality at *p* = 0.05. As shown in Table 3, there was no significant difference in the ABC scores between the two groups before tDCS treatment (*p* > 0.05). After treatment, the behavioral abilities of both groups improved and were statistically significant compared with those before treatment (*p* < 0.05). The comparison between the two groups of patients showed that the atDCS group performed significantly better than the stDCS group, thus indicating that the anode tDCS has a positive effect on the treatment of ASD.

**TABLE 3:** Evaluation results of the ABC scale pre- and post-tDCS in the two groups.

Groups	Pre-tDCS	Post-tDCS	*p*
atDCS	80.21 ± 22.04	59.32 ± 17.30	**<0.001**
stDCS	84.00 ± 20.69	71.39 ± 17.91	**<0.001**
*p*	−2.086	**0.044**	

*Statistical significance is shown in bold font style.*

### Mismatch Negativity Features

The responses were averaged separately for each stimulus type for each subject. To quantify the MMNs, the evoked response to the standard tone was subtracted from the corresponding deviant stimulus response, and the amplitude and latency at peak were measured for all electrodes.

**FIGURE 1:**
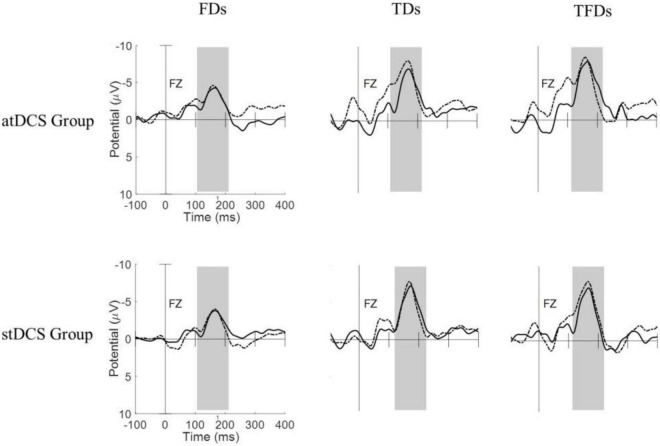
Responses of the three types of deviation stimuli (FDS, TDS, and TFDS) from pre- and post-tDCS states in the two groups of subjects (atDCS group and stDCS group). Dashed line = pre-tDCS, solid line = post-tDCS.

**FIGURE 2:**
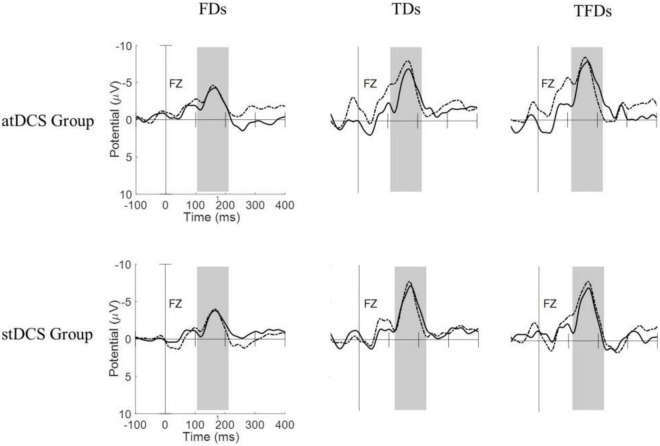
Grand averages of MMNs for all subjects of each group under the three types of deviation stimuli. Dashed line = pre-tDCS, solid line = post-tDCS.

Figure 1 shows the MMN waveforms from different deviant tones in the Fz channel of two typical subjects (one from the atDCS group and the other from the stDCS group). It can be seen from the figure that the MMNs from FDS have lower amplitudes and shorter latencies than with TDS and TFDS. After 4 weeks of treatment, the participants’ MMNs showed an increase in amplitude and decrease in latency, and the average changes in the atDCS group were greater than those in the stDCS group. Figure 2 shows the grand averages of Fz for each group. Similar to Figure 1, the MMN amplitude of the two groups of subjects increased after treatment, but the difference between the groups did not seem to be significant.

**FIGURE 3:**
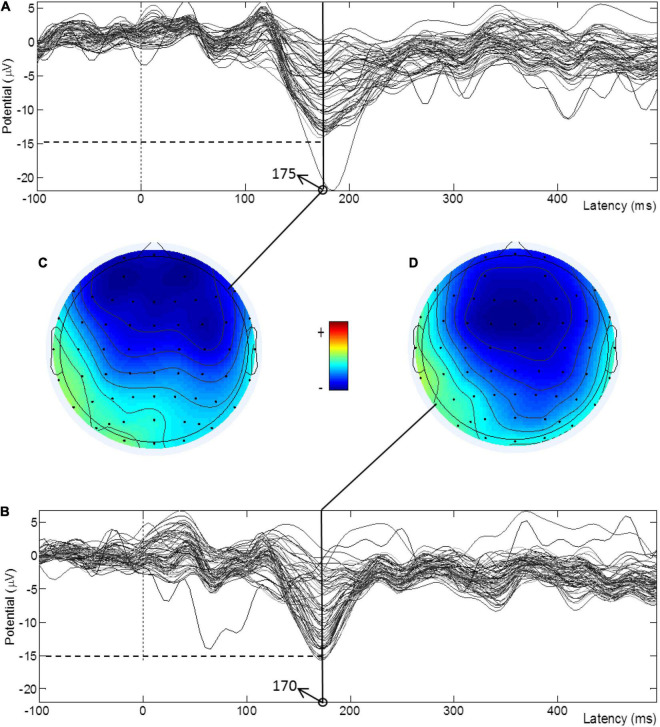
TDS-evoked MMN waveforms of all electrodes pre- and post-treatment for a subject in the atDCS group and the amplitude brain topographies at the peak moments. **(A)** MMN waveform before treatment. **(B)** MMN waveform after treatment. **(C)** Peak amplitude topographic map before treatment. **(D)** Peak amplitude topographic map after treatment.

Figure 3 shows the TD-evoked MMN waveforms of all electrodes pre- and post-treatment for a subject in the atDCS group and the amplitude brain topographies at the peak moments of most electrodes. It can be seen from the MMN waveforms that most of the amplitudes increased after treatment and that the latencies decreased. Figures 3C,D show that the blue area of the brain topographic map increased after treatment, thus indicating that the activation range was enlarged.

Table 4 shows the statistical analysis focused on MMN latency and amplitude in the atDCS and stDCS groups at the Fz electrode measured under the three deviant conditions. There was no statistical difference in the amplitude and latency of all participants’ MMNs when participating in this study. After 4 weeks of treatment, the average MMN amplitude and latency, respectively, increased and decreased for both groups under the three types of deviation stimuli. The improvement in the atDCS group was more significant than that in the stDCS group. The *p*-value in bold font style represents statistical difference (*p* < 0.05). In the atDCS group, all evaluated items were statistically significant, except for the latency of the FDS. The changes in the amplitude of TDS and TFDS and the latency of TDS in the stDCS group were statistically significant. Although the differences in MMN amplitude and latency were not statistically significant, the average MMN latency and amplitude were, respectively, shorter and higher in the atDCS group than in the stDCS group.

**TABLE 4:** Mean amplitudes and latencies of MMN in two groups of children at the Fz electrode.

Deviant type	MMN wave	atDCS group (*n* = 19)	stDCS group (*n* = 18)	*p*
FDS	Pre-tDCS latency (ms)	163.68 ± 5.74	164.44 ± 5.66	0.688
	Post-tDCS latency (ms)	161.05 ± 4.59	162.78 ± 4.28	0.698
	*p*	0.056	0.083	
	Pre-tDCS amplitude (uV)	4.40 ± 0.92	4.19 ± 1.04	0.525
	Post-tDCS amplitude (uV)	4.97 ± 0.78	4.38 ± 1.00	0.051
	*p*	**0.015**	0.454	
TDS	pre-tDCS latency (ms)	171.84 ± 6.91	172.78 ± 5.75	0.658
	post-tDCS latency (ms)	167.63 ± 5.86	169.17 ± 4.62	0.384
	*p*	**0.002**	**0.019**	
	Pre-tDCS amplitude (uV)	8.07 ± 2.09	7.92 ± 1.99	0.826
	Post-tDCS amplitude (uV)	8.86 ± 1.93	8.54 ± 1.81	0.608
	*p*	**0.002**	**0.004**	
TFDS	Pre-tDCS latency (ms)	167.11 ± 6.08	167.22 ± 5.48	0.951
	Post-tDCS latency (ms)	162.37 ± 5.62	165.28 ± 6.06	0.139
	*p*	**0.02**	0.261	
	Pre-tDCS amplitude (uV)	8.41 ± 2.18	8.14 ± 1.92	0.688
	Post-tDCS amplitude (uV)	9.32 ± 2.16	8.83 ± 1.54	0.436
	*p*	**0.011**	**0.031**	

*Statistical significance is shown in bold.*

In addition to Fz, we analyzed the changes in the MMN features of all other leads. Table 5 shows the electrode names with statistical differences pre- and post-tDCS intervention, which can be seen mainly in the prefrontal, temporal, and central areas. The atDCS group had more electrodes with significant differences than the stDCS group. Among the three types of deviated stimuli, TDS have the largest number of differential electrodes, followed by TFDS, and FDS had the lowest number of differential electrodes.

**TABLE 5:** List of electrodes with significant differences (*p* < 0.05) in the amplitude and latency of the three deviation stimuli between the two groups.

Deviant type	MMN features	Electrodes	
		atDCS group	stDCS group
**FDS**			
	Latency	FP1, F3, F1, FC1, C5, C3, T8	AF3, F5, F6, F8, C5, CP5
	Amplitude	F5, Fz, FC1, FCz, CP1, P3, PO3	F1, FT7, T7, TP7, CP4
**TDS**			
	Latency	F1, Fz, F2, FC1, FCz, Cz, C2, CPz, CP6, P6, PO8	Fz, FC3, FC1, C3, P5, PO5, O1
	Amplitude	FP1, AF3, F5, F1, Fz, F4, FC1, FCz, CPz, P4, P6, O2	FPz, FP2, F5, Fz, F2, FC2, CP2, POz, O2
**TFDS**			
	Latency	FP1, FPz, AF4, Fz, F2, FCz, FC2 C2, CP2, CP4	F3, F1, F2, C3, C1, P5, P2, POz
	Amplitude	FP1, F7, F5, F1, Fz, FC1, FC4, CP3, P4, O2	F3, Fz, F2, FC2, C2, CP3

### Correlation Between Aberrant Behavior Checklist and Mismatch Negativity

**FIGURE 4:**
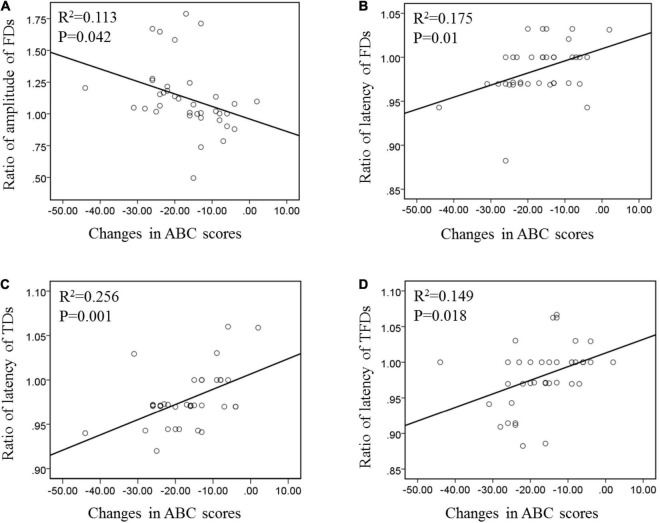
Correlation of the changes between MMN and behavior ability under the three types of deviation stimuli pre- and post-tDCS. **(A)** Correlation between the amplitude of FDS and ABC. **(B)** Correlation between the latency of FDS and ABC. **(C)** Correlation between the amplitude of TDS and ABC. **(D)** Correlation between the amplitude of TFDS and ABC.

The features of ERP components are generally affected by individual differences. This study adopted a special normalization process to accurately reflect the improvement of patients. The ratio of post-tDCS to pre-tDCS was used as an index to evaluate the degree of MMN improvement. The difference between the post-tDCS and pre-tDCS scale scores was used as an indicator of behavioral changes. Figure 4 shows the correlation between the changes in MMN components at the Fz electrode and the changes in behavioral abilities for all subjects under the three types of deviation stimuli. Only results with *p* < 0.05 are shown in Figure 4. The amplitude and latency of the FDS demonstrated a significant linear correlation with the improvement in behavioral ability pre- and post-tDCS (*p* < 0.05). For TDS and TFDS, only the latency of MMN had a significant linear correlation with the improvement in behavioral ability (*p* < 0.05), and there was no statistical relationship between behavioral ability and amplitude (*p* > 0.05).

## Discussion

tDCS is a typical representative of non-invasive brain stimulation (NIBS) that can use a weak current to adjust the activity of cerebral cortex neurons and change the potential of transmembrane neurons, thereby affecting the level of excitability and regulating the firing rate of neurons (Vines et al., 2008). In the current study, the anode tDCS was used to intervene with the DLPFC area of children with ASD, and the effects of behavioral abilities and evoked EEG characteristics were investigated in a controlled study. The results show that 4 weeks of rehabilitation treatment can effectively increase the amplitude of auditory-induced MMN, decrease the latency, and improve behavioral ability. After the treatment, the improvements in participants with anode stimulation were generally greater than those with sham stimulation.

In this study, the anode electrode was placed in the left DLPFC. Abnormalities in the prefrontal area are closely related to symptoms such as attention distribution disorder, weak executive ability, and organization disorder in patients with ASD (Zhou et al., 2020). Dickinson et al. (2016) showed that there is an imbalance between excitation and inhibition in the neural circuits of patients with ASD, particularly in the DLPFC. The results of the current study showed that the behavioral abilities of the two groups of patients significantly improved after treatment. The atDCS group improved more than the stDCS group, thus indicating that the effect of anode tDCS in the DLPFC area can effectively regulate the relationship between neuronal excitation and nerve conduction circuit inhibition. The results of our study reveal that tDCS can improve the coordination ability of the brain and nervous system and have a positive effect on the treatment of ASD.

The MMN reflects the processing of sound differences by the human auditory system. The latency represents the functional state of the auditory sensory pathway, and the amplitude is closely related to the state of the cortex (Näätänen et al., 2007). Relevant studies have shown that the MMN of children with ASD is significantly weaker than that of healthy children (lower amplitude and longer latency), thus indicating that children with ASD may have a weaker response to abnormal environmental stimuli at the auditory level (Vlaskamp et al., 2017; Chien et al., 2018). Impey et al. (2016) used MMN to evaluate the effect of tDCS on the auditory perception of healthy participants, and the results showed that tDCS can enhance the MMN component (increased amplitude and shortened latency). In the current study, the evoked EEG data of the three types of deviated stimuli were collected. As shown in Figure 1, the average amplitude of the FDS was lower than that of the other two deviated stimuli, and this finding might be caused by the difficulty in distinguishing the difference between 1,000 and 1,500 Hz sound stimuli. The latency of the FDS was the shortest among the MMN components of the three types of deviation stimuli. We believe that the time deviation is a process that requires accumulation over time, whereas the frequency deviation can be distinguished instantaneously. Therefore, the MMN evoked by FDS appears to have less latency. The increase in amplitude and decrease in latency suggest functional improvements in neurological pathways and are correlated with ABC score improvements. In the atDCS group, except for the change in latency from FDS, pre-tDCS did not show statistical significance compared with post-tDCS, but the other features improved significantly. The latency amplitude of TDS and TFDS and the latency of TDS in the stDCS group improved significantly after treatment. Overall, the improvement in the atDCS group was higher than that in the stDCS group, but there was no statistical difference in MMN features between the two groups after treatment. Considering that the syndromes of ASD and the neurological deficits that lead to ASD might be complicated, a 4 week treatment might not be enough to cure ASD completely. Therefore, the long-term efficacy of tDCS treatment on ASD should be investigated with precise control of other stimulation parameters in the future. Another important information given in Figure 3 is that after the treatment of anode tDCS, the activation area of the brain increased, thus indicating that tDCS has a positive significance in the neuroregulation of children with ASD; this result also verifies the results of previous studies (Amatachaya et al., 2015; Palm et al., 2016).

Liu et al. (2017) showed that subjects with lower cognitive function scores had a longer ERP latency and a lower amplitude. In the current study, the correlations of the changes between MMN and behavioral ability under these three types of deviation stimuli were analyzed in ASD participants pre- and post-tDCS. The results show that the changes in the amplitudes of the proposed three types of deviant stimuli are linearly related to the changes in the ABC scale scores, thus indicating that MMN features can reflect the behavioral ability of children with ASD and can be used as an objective and quantitative evaluation method. This finding is consistent with the results of Wang et al. (2018), who showed that tDCS can improve the MMN amplitude of patients with impaired consciousness and concluded that MMN is expected to serve as an auxiliary evaluation tool for the treatment effect of tDCS. Only the FD stimulus induced the latency features of MMN and correlated with changes in behavioral ability, thus showing that the response speed of MMN evoked by FDS can better reflect the behavioral ability level in children with ASD.

The current study has some limitations. First, rehabilitation treatment for ASD is generally a long process. Four weeks of tDCS intervention might not be long enough to demonstrate its unique advantages in improving the MMN features of some participants. Second, the pre- and post-tDCS clinical data of subjects may not be sufficient. Follow-up studies with a more comprehensive evaluation of patients, including fMRI and functional near-infrared spectroscopy, are needed to further investigate the effectiveness of tDCS in children with ASD.

## Conclusion

In this study, we utilized tDCS to stimulate the DLPFC area among children with ASD and analyzed the changes in evoked EEG signals and behavioral abilities in the anode and sham stimulation groups. The results showed that the latency of MMN in children with ASD tended to decrease and that the average amplitude tended to increase after stimulation; these features are similar to the MMN features from healthy children. The ABC scale evaluation results show that both tDCS treatment and traditional rehabilitation treatment can effectively improve the behavioral ability of children with ASD, and the improvement is more significant after the use of anode tDCS. The results of the correlation analysis between EEG and the scale show that MMN features have a good correlation with the behavioral ability of children with ASD, particularly the latency features of MMN. Despite the limitations of this study, it is evident that tDCS has positive effects on children with ASD, and the features of MMN can be considered in the assessment of children with autistic behavior ability.

## Data Availability Statement

The raw data supporting the conclusions of this article will be made available by the authors, without undue reservation.

## Ethics Statement

The studies involving human participants were reviewed and approved by the Ethics Committee of Tianjin Union Medical Centre, Tianjin, China. Written informed consent to participate in this study was provided by the participants’ legal guardian/next of kin.

## Author Contributions

CS, YZ, and CW contributed to study design and patient recruitment. ZZ, RT, and WZ contributed to data collection. CS and LC contributed to data analysis, data interpretation, and manuscript preparation. JD and YZ contributed to data interpretation and critically revised. All authors contributed to the article and approved the submitted version.

## Conflict of Interest

LC was employed by China Electronics Cloud Brain (Tianjin) Technology Co., Ltd., Tianjin, China. The remaining authors declare that the research was conducted in the absence of any commercial or financial relationships that could be construed as a potential conflict of interest.

## Publisher’s Note

All claims expressed in this article are solely those of the authors and do not necessarily represent those of their affiliated organizations, or those of the publisher, the editors and the reviewers. Any product that may be evaluated in this article, or claim that may be made by its manufacturer, is not guaranteed or endorsed by the publisher.
